# A Study of the Effect of Heat-Treatment on the Morphology of Nafion Ionomer Dispersion for Use in the Passive Direct Methanol Fuel Cell (DMFC)

**DOI:** 10.3390/membranes2040841

**Published:** 2012-12-06

**Authors:** Ting Yuan, Haifeng Zhang, Zhiqing Zou, Sufia Khatun, Daniel Akins, Yara Adam, Sophia Suarez

**Affiliations:** 1 Shanghai Advanced Research Institute and Shanghai Institute of Microsystem and Information Technology, Chinese Academy of Sciences, Shanghai 201210, China; E-Mails: yuanting0130@yahoo.com.cn (T.Y.); yangh@sari.ac.cn (Z.Z.); 2 Physics Department, Hunter College of the City University of New York, New York, NY 10021, USA; E-Mail: sufia77@hotmail.com; 3 CASI and Chemistry Department, The City College of the City University of New York, New York, NY 10031, USA; E-Mail: akins@sci.ccny.cuny.edu; 4 Physics Department, Brooklyn College of the City University of New York, Brooklyn, NY 11210, USA; E-Mail: yara.adam@gmail.com

**Keywords:** Nafionionomer, aggregation, membrane electrode assembly, catalyst utilization, direct methanol fuel cell, NMR, viscosity

## Abstract

Aggregation in heat-treated Nafion ionomer dispersion and 117 membrane are investigated by ^1^H and ^19^F Nuclear Magnetic Resonance (NMR) spectra, spin-lattice relaxation time, and self-diffusion coefficient measurements. Results demonstrate that heat-treatment affects the average Nafion particle size in aqueous dispersions. Measurements on heat-treated Nafion 117 membrane show changes in the ^1^H isotropic chemical shift and no significant changes in ionic conductivity. Scanning electron microscopy (SEM) analysis of prepared cathode catalyst layer containing the heat-treated dispersions reveals that the surface of the electrode with the catalyst ink that has been pretreated at *ca.* 80 °C exhibits a compact and uniform morphology. The decrease of Nafion ionomer’s size results in better contact between catalyst particles and electrolyte, higher electrochemically active surface area, as well as significant improvement in the DMFC’s performance, as verified by electrochemical analysis and single cell evaluation.

## 1. Introduction

Direct methanol fuel cells (DMFCs) have been widely developed and explored in the last few decades [1,2,3]. Although there are some issues, such as durability, and cost of raw materials, micro-DMFCs are considered to be the most promising alternative power source for portable devices [2,4]. To achieve these ends, tremendous research effort has been devoted to the development of novel membrane electrode assemblies (MEAs) in order to overcome the remaining drawbacks (e.g., sluggish kinetics reactions of the electrode and relatively low power density) of DMFCs [1,2,3,4].

The MEA as the core of a DMFC plays an important role in the cell’s performance [2]. It consists of a proton exchange membrane sandwiched between catalyst layers. In order to improve the performance of MEAs, many investigations have focused on the use of novel nanosized catalysts with high catalytic activity [5,6,7]. However, it is well known that the electrochemical reactions take place at the three-phase boundary zone consisting of catalyst, reactants and electrolyte. Every effective reaction active site is composed of catalyst particles and Nafion (^®^DuPont) ionomer. Catalyst particles as the active catalytic sites and electron conductor have to connect with the proton conducting Nafion ionomer to ensure high catalyst utilization and better cell performance. Therefore, it is very important to extend the three-phase reaction zone. Much effort has been dedicated at maximizing the contact area between the catalyst particles and electrolyte, such as preparing catalyst layer from an ink with Nafion ionomer [8].

However, some studies have shown that the main obstacle to maximizing the contact area between the catalyst and electrolyte are the significant differences in size of the carbon and catalyst particles, and Nafion micelles, as well as the tendency for the latter to aggregate [9]. The Nafion ionomer have different aggregate sizes and arrangements. The most common model has the Nafion fluorocarbon molecules aggregating into “primary” compact cylinder particles with ionic side chains around the periphery of the cylinders. These primary cylinders can then form “secondary” aggregates through the electrostatic interactions of the peripheral ionic groups [10]. It has been reported that two aggregation processes of Nafion molecules occur in the alcohol/water mixture. Xie *et al.* found that Nafion aggregated more in water than in n-propyl alcohol (NPA)/water [11]. This behavior is believed to be responsible for differences found in fuel cell performance for catalyst layers prepared from the two different dispersion media [11]. Wang *et al.* have investigated the effect of Nafion ionomer aggregation in solution on the structure of the cathode catalyst layer of a DMFC [12]. They found that the large aggregation particles in Nafion aqueous solution were significantly suppressed by the addition of NaOH to the solution, resulting in a higher electrochemical surface area and better performance for a DMFC. Wu *et al.* found that the ionomer deposits onto multiwall carbon nanotubes (MWCNTs) after the addition of MWCNTs to anode catalyst layer, resulting in the formation of pathways for protons and enhancing proton conductivity [13].

We have studied the aggregation state of Nafion ionomer in the solution under different temperature treatments by dynamic light scattering (DLS) [14,15,16]. The results, described herein for different heat treatments (at 25, 50 and 80 °C), show that the Nafion aggregate sizes in the suspension decrease and that the agglomerate particle size distribution becomes narrower with the increase in heat-treatment temperature until nearly monodispersed ionomers are obtained at *ca.* 80 °C. In order to extend our finding to practical applications, we have investigated the effect of Nafion aggregation in the catalyst layer on the performance of a direct formic acid fuel cell as well as a DMFC.

In this present work, we extended the previous study [16] by examining the effect of heat-treatment on ionomer dispersion using ^19^F NMR, and AC Impedance spectroscopy. NMR spectra, spin-lattice relaxation times (*T*_1_) and self-diffusion coefficient measurements (D-^19^F for dispersions only) were determined for 25, 50, and 80 °C heat-treated membrane, and ionomer dispersion without added Pt and C. We have also examined the performance of a passive DMFC with decreased Nafion aggregate size within the cathode catalytic layer. The cathode catalytic layer is responsible for the transport of both oxygen and water, therefore improving its performance is crucial to the efficient operation of the DMFC. Additionally, the structure of the cathode catalyst layer was characterized by scanning electron microscopy (SEM) and its performance was investigated through electrochemical analysis and a single cell evaluation.

## 2. Results and Discussions

### 2.1. NMR, Viscosity, and Membrane Ionic Conductivity Results

#### 2.1.1. Nafion Ionomer Solutions

**Figure 1:**
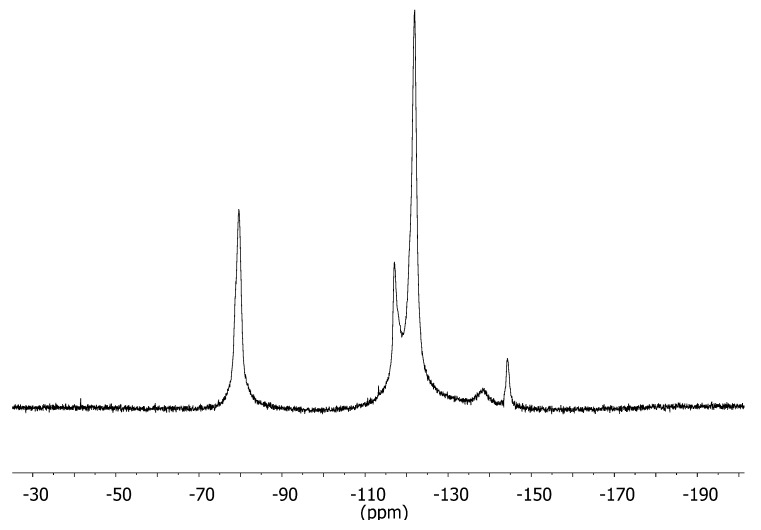
^19^F Nuclear Magnetic Resonance (NMR) spectrum of representative Nafion ionomer in static fluorine-free probe at 25 °C.

^19^F NMR spectra (obtained using a static fluorine free-probe) for the 25, 50, and 80 °C heat-treated Nafion ionomer solutions were identical, with peaks at approximately −80, −120, and −140 ppm as shown in Figure 1. The assignments of these peaks have been reported [17]. No difference was observed in the ^19^F chemical shifts, which suggests there was no significant change in the fluorine’s local environment.

The ^19^F *D* and *T*_1_ values for the solutions were obtained in the Doty diffusion probe. For *D* measurements, peaks located at about −80 and −120 ppm as shown in Figure 1 were observed for the whole ionomer system. For *T*_1_ measurements, a broad multiple component spectrum that spanned the frequency range of −50 to −200 ppm was observed for each dispersion, partly due to probe fluorine background. The values (for both *D* and *T*_1_) given in Table 1 are the average of all peaks observed. *T*_1_ values were almost identical for all three solutions. 

This suggests the fluorines are either well shielded, or their rapid motion averages the dipole-dipole interactions between them and the protons. This is supported by the spectra, which showed no difference between the solutions. *D* values were observed to increase with increasing treatment temperature; however, there was a greater increase between the 80 and 50 °C samples compared to that between the 50 and 25 °C. In order to interpret the increased diffusion coefficients in heat treated solutions in terms of changes in ionomer aggregation, it is necessary to know solution viscosities.

**Table 1:** ^19^F NMR *T*_1_ and *D* values, Viscosities and calculated radii for Nafion ionomer solutions heat-treated at 25, 50, and 80 °C.

Sample Name	Heat-Treatment Temperature (°C)	Nucleus (^19^F)			
		*T*_1_ (s) (±0.05)	*D* (×10^−11^ m^2^/s) (±0.1)	Viscosity *η* (×10^−3 ^Pa s)	Radius (×10^−9^ m)
A	25	0.92	3.2	1.9	3.6
B	50	0.89	3.6	2.2	2.8
C	80	0.90	4.4	2.4	2.1

Viscosity values determined for the dispersion are also shown in Table 1. The trend is an increase in viscosity with increasing treatment temperature. The Stokes-Einstein equation (shown below) was used to determine hydrodynamic radii of the Nafion aggregates, which are also provided in Table 1.

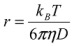

Here, *r* is the radius of the molecule, *k*_B_ = Boltzmann constant (1.38 × 10^−23^ m^2 ^kg s^−2^ k^−1^), *T* is the temperature in Kelvin, *η* is the viscosity of the solution, and the factor of 6 is from the approximation that the particle interacts strongly with the solvent molecules. The resultant trend is a decrease in radius with increasing treatment temperature. The fact that the ionomer aggregates are not spherical and hence do not strictly conform to the Nernst-Einstein equation will be discussed in more detail later.

*T*_1_ measurements provide information on the motions occurring at the inverse of the Larmor frequency timescale (µs), while *D* measurements look at motions on a larger timescale (ms–s). The *T*_1_ values were almost identical, but there were significant differences between the *D* values. This finding, coupled with the viscosity results supports possible morphological changes resulting from heat-treatment that enhance the long-range translational motion.

#### 2.1.2. Nafion 117 Membrane

To determine if this heat treatment effect modified the nature of the membrane form of Nafion, ^1^H NMR spectra and *T*_1_ values were determined for the untreated and heat-treated Nafion 117 membranes. The spectrum for all membranes consisted of a single peak. Linewidths (FWHM) for all the samples were similar to that of the untreated Nafion 117, with values of ~100 Hz. Deconvolution of the spectra using combined Gaussian and Lorentzian lineshapes (Peak Fit Software) revealed multiple components for each sample. The isotropic chemical shift (peak position) was dependent on treatment temperature, increasing to higher frequency with increasing treatment temperature. 

The ^1^H *T*_1_ values for the membranes (as shown in Table 2) showed similar values for all heat-treated samples. Water uptake results for all three samples were comparable to that of untreated Nafion 117 (27%), with values of 23%, 24% and 28% for the 25, 50 and 80 °C, respectively. 

**Table 2:** ^1^H NMR *T*_1_, and ionic conductivity values for Nafion 117 membrane heat-treated at 25, 50, and 80 °C.

Sample Name	Heat-Treatment Temperature (°C)	Nucleus (^1^H)	
		*T*_1 _(s)	Ionic Conductivity (S/cm)
Nafion 117 membrane (ref.)	–	0.22	0.062
A	25	0.25	0.079
B	50	0.20	0.079
C	80	0.20	0.077

Ionic conductivity values were also determined for the membranes and are given in Table 2. All three heat-treated membranes had slightly higher ionic conductivity compared to the untreated membrane. There was however no significant difference between the values.

^19^F NMR data for the ionomer solutions shows heat-treatment enhances the long-range translational motion of the chains or micelles. Additionally, it suggests a local environment mediated by rapid short-range motions, and reduced restrictions in the long-range translational motion. Several studies have shown that Nafion form fibers or rod-like structures in aqueous solutions and in membrane form [18,19]. These rods are suggested as having the SO_3_^−^ groups externally running along the rod. They are reported as being heat and concentration dependent. One study on Nafion membranes [19] reported the onset of fiber formation at 60 °C, and that it continues to undergo morphological changes leading to smaller diameter fibers at 80 °C, as well as narrower size distribution. A study on Nafion solutions [18] reported these structures as occurring for low concentrations (>0.2 mg/mL), and also suggests that the structures change from rigid rod-like to more loosely and possibly entangled structures with increasing Nafion concentrations. A transition to a thinner fiber structure in heat treated solutions is consistent with the diffusion and viscosity results, which implies smaller particle size. Without specific knowledge of the fiber aspect ratio, which could be used to input a particle shape correction to the Nernst Einstein result, the ionic radii listed in Table 1 are not true measures of aggregate size, but the trend toward smaller size with temperature treatment is clear. In addition, if these structures are in fact present, then this could cause increase de-shielding of the protons through the external –SO_3_^−^ groups, while leaving the fluorine local environment unaffected, which is what the NMR results (shift in ^1^H spectra to higher frequency with increasing heat-treatment temperature) supports. 

### 2.2. The Distribution of Aggregates in the Catalytic Ink and Catalyst Layer

**Figure 2:**
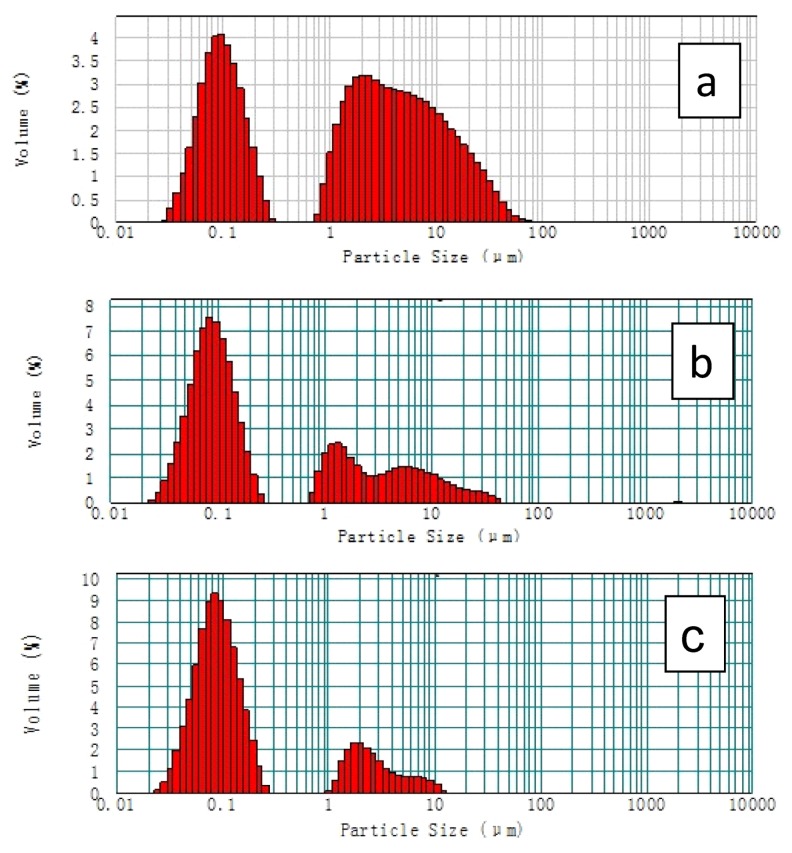
Particle size distributions of catalyst inks pre-heated at (**a**) 25 °C; (**b**) 50 °C and (**c**) 80 °C.

Particle size distributions in the catalyst ink prepared at various temperatures are shown in Figure 2. It is clear that the catalyst ink prepared at the high temperature (80 °C) forms a greater concentration of smaller agglomerates than those prepared at the lower temperatures. For the catalyst ink pre-heated at *ca.* 25 °C, the particle size distribution was mainly in the region of *ca.* 0.026 to 0.316 µm and *ca.* 0.724 to 52.481 µm. This data is shown in Table 3. 

**Table 3:** Particle size distributions and electrochemical characterizations of Pt/C electrode with catalyst ink pretreated at different temperatures.

Pretreated temperature (°C)	Ratio of small particles	ECSA_H _(m^2^ g^−1^)	ECSAco (m^2^ g^−1^)	I (0.85V) (mA cm^−2^)	MP (mW cm^−2^)
25	36.7%	29.1	52.7	0.86	31.7
50	65.5%	39.1	58.2	1.09	34.4
80	79.9%	51.3	63.7	1.45	39.8

About 63.3% of aggregates size ranged from *ca.* 0.724 to 52.481 µm. Clearly, most of aggregates formed from Pt/C particles and Nafion ionomers existed as large aggregated particles at room temperature. When the catalytic ink was pre-heated at *ca.* 50 °C, many large particles broke up to form smaller ones, with only 34.5% of aggregate size ranging from *ca.* 0.724 to 45.7 µm and 65.5% from *ca.* 0.023 to 0.275 µm. At 80 °C, 79.9% of aggregate size ranged from *ca.* 0.023 to 0.275 µm and 20.1% from *ca.* 1.096 to 13.183 µm. Particles larger than 14 µm completely disappeared. Obviously, the large agglomerates formed from Pt/C particles and Nafion ionomers are dissociated to form small ones at elevated temperatures.

**Figure 3:**
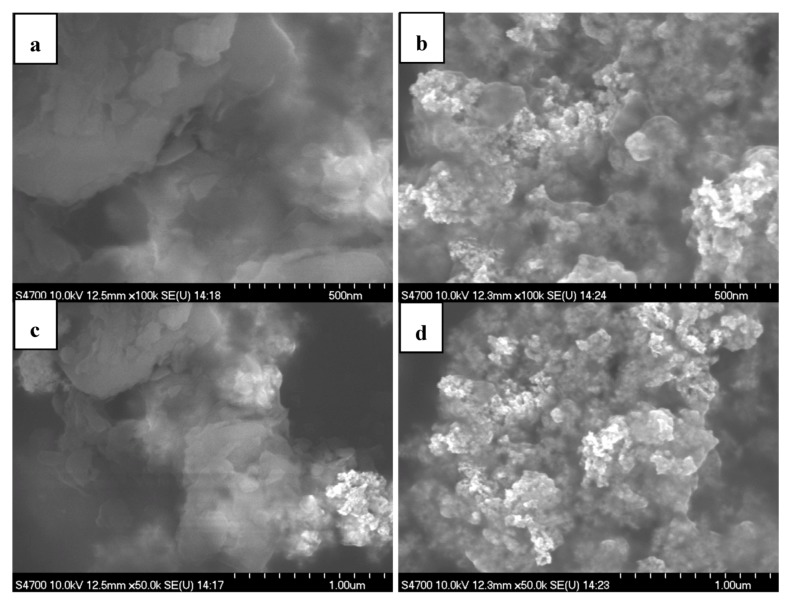
Scanning electron microscopy (SEM) images of two Pt/C catalytic electrodes using the catalyst inks pre-heated at (**a**,**c**) 25 °C and (**b**,**d**) 80 °C, respectively.

Figure 3 shows typical SEM images of two Pt/C catalytic electrodes made with catalyst inks pre-heated at *ca.* 25 and 80 °C, respectively. It can be seen that the electrode made from the ink heat-treated at *ca.* 80 °C exhibits a compact and uniform distribution, while larger aggregates exist on the surface of the electrode when the catalytic ink was pre-heated at *ca.* 25 °C. The difference in morphologies of two catalytic electrodes might be attributed to the different aggregation size of Nafion ionomers pre-heated at different temperatures, suggesting that the catalyst particles are assessable in the small ionomers.

### 2.3. Electrochemistry and Electrocatalysis of Pt/C Catalytic Electrode with Tuned Aggregates Size for the Oxygen Reduction

**Figure 4:**
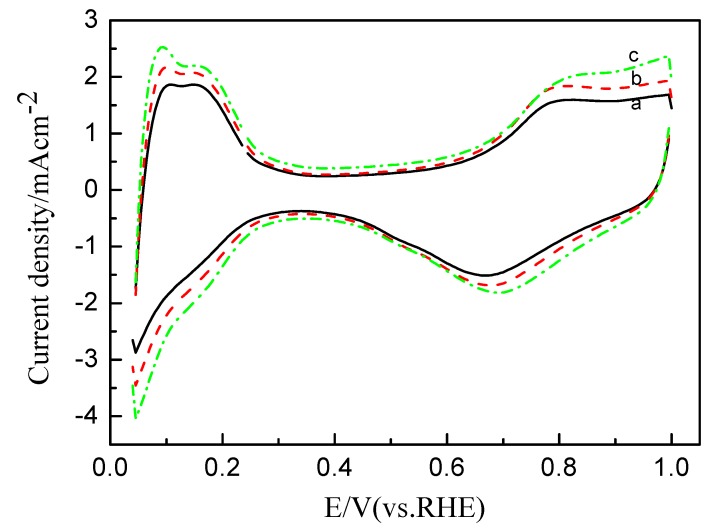
CVs of Pt/C catalytic electrodes with the catalyst ink pre-heated at (**a**) 25 °C; (**b**) 50 °C and (**c**) 80 °C in 0.1 M HClO_4_ at a scan rate of 50 mV s^−1^.

**Figure 5:**
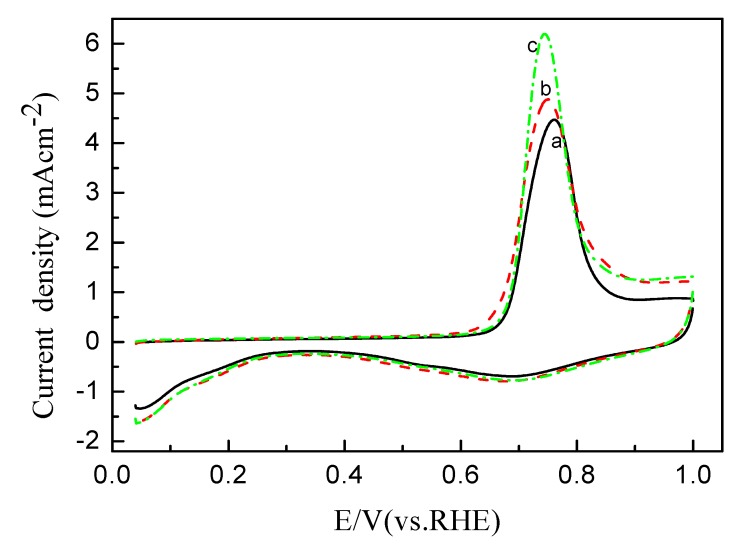
CO-stripping voltammograms of Pt/C catalytic electrodes with the catalyst ink pre-heated at (**a**) 25 °C; (**b**) 50 °C and (**c**) 80 °C in 0.1 M HClO_4_ at a scan rate of 20 mV s^−1^.

The electrochemical properties of Pt/C electrodes with ink preheated at different temperature were first examined by cyclic and CO-stripping voltammetry, as shown in Figure 4 and Figure 5, respectively. Stable voltammograms were obtained after 30 cycles. Three distinct potential regions were observed in the voltammograms: The hydrogen adsorption/desorption region between 0.05 and 0.31 V, the double-layer region between 0.31 and 0.60 V, and the surface oxide formation/reduction region (>0.60 V) [20]. Both the H region area and CO_ad_ oxidation area were used to calculate the electrochemically active surface area (ECSA) listed in Table 3. As can be seen, the ECSA_CO_ is larger than ECSA_H_; however, both have the same trend and are essentially proportional to the particle size distribution.

**Figure 6:**
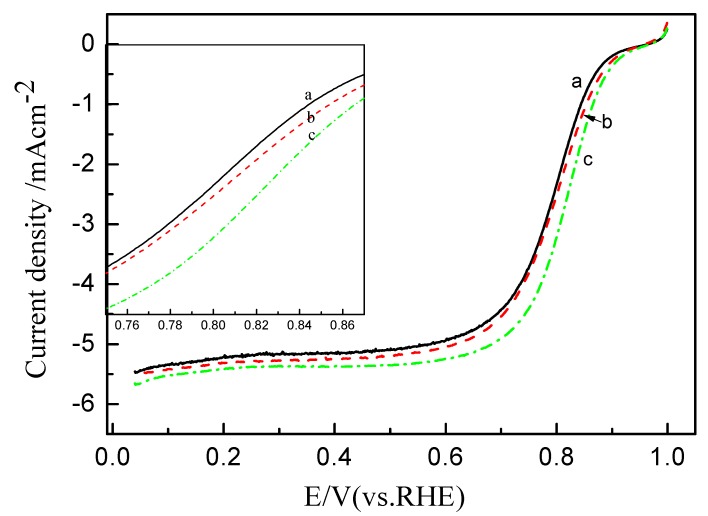
Linear scan voltammograms (LSVs) of Pt/C catalytic electrodes with the catalyst ink pre-heated at (**a**) 25 °C; (**b**) 50 °C and (**c**) 80 °C in 0.1 M HClO4 saturated with pure O_2_ at a scan rate of 5 mV s^−1^.

Oxygen reduction reaction (ORR) polarization curves for Pt/C electrode with ink preheated at different temperatures in oxygen-saturated 0.1 M HClO_4_ at room temperature are presented in Figure 6. From the figure, the ORR on the catalyst is diffusion-controlled when the potential is less than 0.7 V and is under mixed diffusion kinetics control in the potential region between 0.7 and 0.85 V. For the sake of clarity, a close-up of this region is shown in the inset of Figure 6. For the pretreatment temperature used, the ORR activity of the Pt/C electrode increases in the order: 80 °C > 50 °C > 25 °C. At a given potential of 0.85 V, the current densities of Pt/C electrodes pretreated at *ca.* 25, 50, 80 °C are *ca.* 0.86, 1.09 and 1.45 mA cm^−1^, respectively, clearly indicating that the aggregation state of Nafion ionomer plays a significant role in the improvement of the ORR activity.

### 2.4. Performance Comparison of the Passive DMFCs with Cathode Catalyst Ink Pre-Heated at Various Temperatures

**Figure 7:**
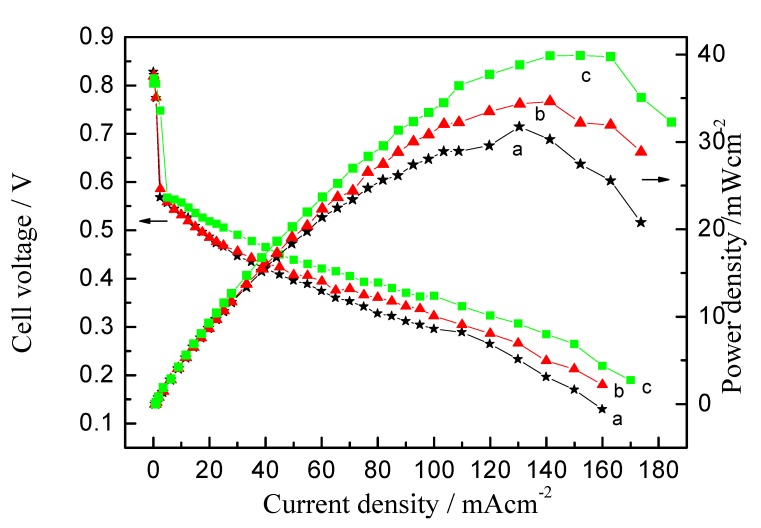
Performance comparison of the passive direct methanol fuel cells (DMFCs) with cathode catalytic layer made from the ink pre-heated at (**a**) 25 °C; (**b**) 50 °C and (**c**) 80 °C.

To evaluate the effect of Nafion aggregation behavior on the cell’s performance, a performance comparison of the passive DMFCs with cathode catalytic layer made from the ink pre-heated at *ca.* 25, 50 and 80 °C is given in Figure 7. It is clear that the open circuit voltage of the passive DMFC increases with the pre-heating temperature for the catalyst ink. For the cathode catalyst ink pre-heated at *ca.* 25 °C, the maximum power density of a passive DMFC is *ca.* 31.7 mW cm^−2^. However, when the cathode catalyst ink was pre-heated at *ca.* 50 °C, a maximum power density *ca.* 34.4 mW cm^−2^ was obtained. With further increase to *ca.* 80 °C for cathode catalyst ink, the maximum power density is *ca*. 39.8 mW cm^−2^. These findings indicate that the power density of the passive DMFC can be improved significantly by pre-heating the cathode catalyst ink.

## 3. Experimental

### 3.1. NMR, Viscosity and IS Measurements of Nafion 117 Membrane and Ionomer Solutions

^19^F NMR measurements were performed on as-received Nafion solution (Aldrich, 5 wt % in lower aliphatic alcohols and water) and ^1^H NMR and Impedance Spectroscopy (IS) measurements were also done on Nafion 117 membrane, heat-treated at 25, 50 and 80 °C. NMR Measurements were performed using both a 300 MHz Varian Direct Digital Drive (^19^F) and a Varian Unity Plus (^1^H) spectrometers with 7.1 Tesla superconducting magnets. For ^19^F measurements, a fluorine free static broadband probe was used to obtain NMR spectra, and a 5mm Doty dual frequency diffusion probe was used for the self-diffusion (*D*) measurements. ^1^H measurements were obtained on a Varian four-frequency 5 mm probe. NMR spectra were obtained from the Fourier Transform of single π/2 pulses. *D* values were obtained by the Pulsed Gradient Spin Echo (PGSE) technique [21], and gradient values g ranged from 0.5 to 210 T/m. Spin-lattice relaxation times (*T*_1_) were determined by the Inversion Recovery (*π*-*τ*-*π*/2-acquire) pulse sequence. *D* and *T*_1_ measurements were performed at 25 °C. Water and an aqueous solution of LiTf (lithium triflate) were used as references.

Nafion 117 membranes were initially heat treated at 25, 50 and 80°C for 1 hour, then hydrated in distilled water for four days. Strips (3 mm × 25 mm) of the hydrated membrane were cut and placed into 5 mm OD NMR tubes, which were then capped and sealed with parafilm to reduce moisture loss over the course of the measurement. In-plane ionic conductivities were determined by AC Impedance Spectroscopy over the frequency range of 0.01–10^5^ Hz, using a Solartron SI 1260 Impedance Phase Gain Analyzer with the SI 1287 Electrochemical Interface. Samples were sealed in plastic bags to prevent moisture loss during the measurement. Gold foil was used as the electrodes and measurements were performed at 25 °C.

Viscosity measurements were performed on the ionomer solutions using the Gilmont falling ball method. *V* iscosities were obtained by the following equation:

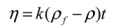

where *η* is the viscosity of liquid, *k* is the constant for the viscometer and stainless steel ball combination, which was determined experimentally using reference solvents of known viscosity, *ρ_f_* is the density of the steel ball, *ρ* the density of the liquid, and *t* the time it takes for the ball to fall in the viscometer. Measurements were obtained at 25 °C.

### 3.2. Measurement of Nafion Aggregation in Catalyst Ink

To prepare the Pt/C catalyst ink, 10 mg of Pt/C catalysts (HiSpec 9100, Johnson Matthey), 0.5 mL of as-received Nafion solution (Aldrich, 5 wt % in lower aliphatic alcohols and water) and 2.5 mL of ultrapure water were mixed ultrasonically to form a suspension. The mixture was heated for 1 h at 25, 50 or 80 °C, followed by further ultrasonification for a selected period of time. The particle distribution of aggregates formed between Pt/C nanoparticles and Nafion ionomers was examined by a Mastersizer 2000 particle size analyzer (Malvern, Inc.) at room temperature.

### 3.3. Characterization of Cathode Catalyst Layer

The surface morphology of a Pt/C catalytic electrode was characterized by scanning electron microscopy (SEM) using a Hitachi S-4700 microscope operated at an accelerating voltage of 20.0 kV.

### 3.4. Electrochemical Characterizations of Cathode Catalyst Layers

The catalyst ink was prepared as mentioned above. A measured volume (*ca.* 3 µL) of this ink was transferred via a syringe onto a freshly polished glassy carbon disk (GC, 3 mm diameter). After the solvent was evaporated overnight at room temperature, the electrode was used as the working electrode. Each electrode contained *ca.* 85 µg cm^−2^ of the metal. Electrochemical measurements were performed using a CHI 730B Potentiostat and a conventional three-electrode electrochemical cell. The counter electrode was a glassy carbon plate, and a saturated calomel electrode (SCE) was used as the reference electrode. All potentials, however, are referenced with respect to the reversible hydrogen electrode (RHE). The real surface areas of all the catalysts were determined by hydrogen desorption and CO_ad_ oxidation in CO stripping voltammetry. The oxidation charges of monolayer adsorption of CO and hydrogen on Pt surface are assumed to be 420 and 210 µC cm^−2^, respectively [18]. High purity nitrogen or oxygen was used for deaeration of the solutions. During the measurements, a gentle gas flow was kept above the electrolyte. The electrolyte was 0.1 M HClO_4_.

### 3.5. Single Cell Test

A slurry which consisted of Vulcan XC-72R carbon and PTFE (20 wt %) was coated onto the carbon paper (TGPH-060, 20 wt % wet-proofing by PTFE, Toray) to form the cathode diffusive layer. The XC-72R carbon loading was *ca.* 2 mg cm^−2^. The cathode catalysts used in this work were Pt black (HiSpec 1000, Johnson Matthey) and carbon-supported Pt with a Pt loading of 60 wt % (HiSpec 9100, Johnson Matthey). Catalyst ink was prepared by dispersing appropriate amount of catalyst and 5 wt % Nafion solution (Aldrich) into a mixture of isopropyl alcohol and Millipore water with a volume ratio of 1:1, and then different samples were heated for 1 h at 25, 50, 80 °C. Subsequently, the catalyst ink was sprayed onto the diffusion layer. The Nafion ionomer loading was 20% and the metal loading was 6 mg cm^−2^ for the cathode.

The anode was fabricated in the same way as the cathode, but the carbon powder in the diffusive layer was multi-walled carbon nanotubes at *ca.* 1 mg cm^−2^ and there was no wet-proofing in the anode carbon paper. The anode catalyst was Pt-Ru black with an atomic ratio 1:1 (HiSpec 6000, Johnson Matthey). The Nafion ionomer loading was 15 wt % and the metal loading was 4 ± 0.2 mg cm^−2 ^for the anode.

The MEAs were fabricated by hot-pressing both anode and cathode on both sides of a pretreated Nafion 117 membrane at 130 °C and 6 MPa for 3 min. Nafion 117 membrane (DuPont) was pretreated as mentioned in previous works [22].

## 4. Conclusions

Heat-treatment was observed to have a greater effect on the Nafion ionomer dispersion than the membrane. For the ionomer dispersion, the results were a decrease in particle sizes and an enhancement of the long-range translational motions of the backbone chains or micelles with increasing treatment temperature. The membranes show increase in the isotropic chemical shift with increasing heat-treatment temperature, suggesting an increase de-shielding of the protons possibly due to the SO_3_^−^ groups. This increasing de-shielding could affect the long range translation motion of the protons. The particle sizes of the aggregates formed from Pt/C particles and ionomers in the catalyst ink decrease with the increase in pre-heated temperature, thus leading to a better dispersion and enhanced contact between Pt/C particles and Nafion ionomers. As a result, the electrode prepared with catalyst ink pre-heated at 80 °C achieved a higher ESA and better performance for a DMFC than that prepared with ink pre-heated at room temperature. The improvement can be attributed to the fact that the large Nafion aggregation ionomers are dissociated to form smaller ones at elevated temperatures, which facilitates better contact between catalyst particle and Nafion ionomer. 
